# Sex and Location Differences in Verification Status of Physician-Held Social Media Platform Accounts

**DOI:** 10.1001/jamanetworkopen.2022.25671

**Published:** 2022-08-08

**Authors:** Deborah Rupert, Kanan Shah, Brian Chen, Avital Y. O’Glasser, Michael Schiml, Shikha Jain, Fumiko Chino

**Affiliations:** 1 Medical Scientists Training Program, Stony Brook University, Stony Brook, New York; 2 New York University Grossman School of Medicine, New York, New York; 3 Department. of Computer Science and Engineering, P.C. Rossin College of Engineering and Applied Science, Lehigh University, Bethlehem, Pennsylvania; 4 Department of Medicine, Division of Hospital Medicine, Department of Anesthesiology and Perioperative Medicine, Oregon Health and Science University, Portland; 5 Case Western Reserve University, Cleveland, Ohio; 6 Department of Medicine, Division of Hematology Oncology, University of Illinois at Chicago, Chicago, Illinois; 7 Department of Radiation Oncology, Memorial Sloan Kettering, New York, New York

## Abstract

This cross-sectional study investigated the association of user sex and location with verification of physician-held social media accounts.

## Introduction

Physicians use social media, including Twitter, to interact with colleagues, amplify career developments, broadcast research, and combat medical misinformation.^1,2,3,4^ Twitter verification indicates that an account is “authentic, notable, and active,” adding a degree of validity. We evaluated characteristics of physician-held verified Twitter accounts (PHAs), hypothesizing that historically marginalized demographic characteristics would be underrepresented.

## Methods

This cross-sectional study used publicly available data and was exempt from ethical review (eAppendix in the Supplement). We accessed a cross-sectional sample of Twitter-verified users via the official @verified account, categorizing accounts by location (US, international) and sex (men, women) and assessed account metrics: date of creation and follower (accounts following the user) and following (accounts the user follows) counts. See eAppendix in the Supplement for description of how sex was determined. We used nonparametric, 2-tailed, unpaired *t* tests to compare metrics for men vs women and US vs international users.

## Results

Among 356 720 verified accounts, there were 779 PHAs. Of 757 verified PHAs with confirmed user sex, there were 535 (70.7%) men and 222 (29.3%) women. Men did not have a significantly different mean (SD) number of follower accounts (92 808 [414 639] accounts vs 28 672  [62 603] accounts; *P* = .06) or accounts they were following (3340 [43 224] accounts vs 2157 [6323] accounts; *P* = .22). After normalization to following size (follower-to-following ratio [FFR]), men had 3.2-fold more mean (SD) followers per account followed (251.2 [1007.0] followers vs 76.4 [251.3] followers; *P* = .06), but this difference was not statistically significant (Figure). After normalizing FFRs to the time accounts were active, men acquired significantly more mean (SD) new followers per person followed per day (0.09 [0.41] followers vs 0.04 [0.14] followers; *P* = .007).

**Figure.**
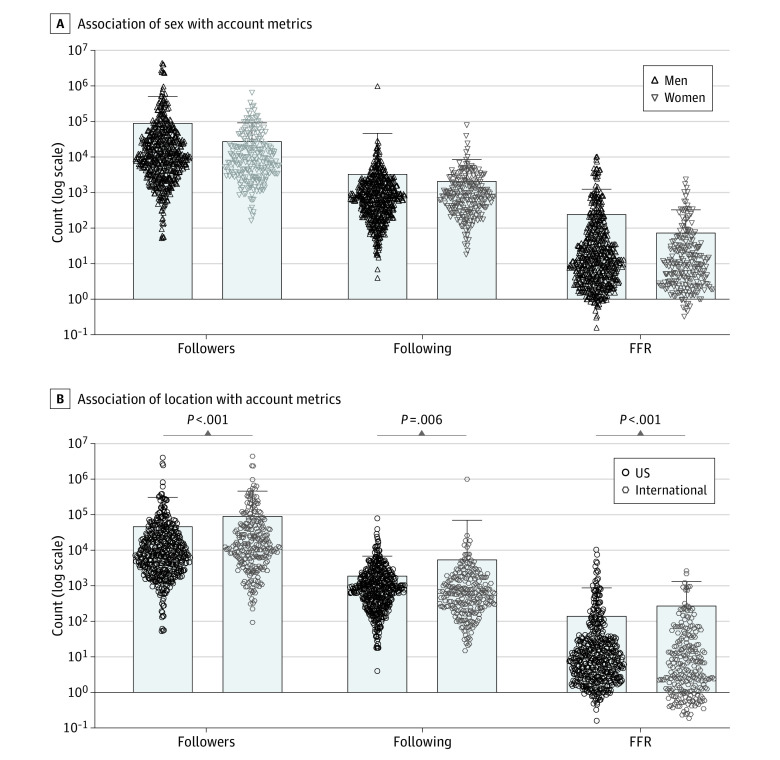
Association of Sex and Location With Account Metrics Bars indicate means; dots and triangles, individual data points; FFR, follower-to-following ratio; whiskers, SDs.

Of 712 confirmed user locations, 466 locations (65.4%) were US based and 246 locations (34.5%) were international. US users had a lower mean (SD) number of follower accounts (48 140 [261 026] accounts vs 94 471 [368 411] accounts; *P* < .001) and accounts they were following (1950 [4936] accounts vs 5604 [63 927] accounts; *P* = .006). US users had significantly lower mean (SD) FFRs (146.6 [732.4] vs 281.2 [1042]; *P* < .001). After normalization to account length, international users had more mean (SD) new followers per person followed per day (0.05 [0.31] followers vs 0.10 [0.43] followers; *P* < .001) (Table).

**Table.** Physician-Held Twitter Account Metrics by Sex and Location

Category	Accounts, No. (%) (N = 779)	Mean (SD)^a^		
		Followers	Following	FFR
Sex (n = 757)				
Men	535 (70.7)	92 808 (414 639)	3340 (43 224)	251.3 (1007.0)
Women	222 (29.3)	28 672 (62 603)	2157 (6323)	76.4 (251.3)
Location (n = 712)				
US	466 (65.4)	48 140 (261 026)	1950 (4936)	146.6 (732.4)
International	246 (34.5)	94 471 (368 411)	5604 (63 927)	281.2 (1042.0)

Abbreviation: FFR, follower-to-following ratio.

^a^ Mean (SD) follower and following counts and FFR provided for each category.

## Discussion

Social media has become part of a physician’s professional and public-facing profile. Verification validates and boosts that status and may have important implications for patient engagement and academic promotion based on digital scholarship. This cross-sectional study found that women physicians held fewer verified PHAs, with lower growth rates. These findings build on prior work showing that men and women used Twitter approximately equally yet women had less reach,^5^ and our results suggest that verification may be unequally distributed. This may suggest that women’s accounts fail to meet a verification threshold (including being in the top 0.05% follower or mention count by location). However, our finding that international physicians, despite higher FFRs, had fewer verified accounts suggests that this is unlikely. Twitter’s verification process lacks transparency, and verification requests are reviewed case by case to assess whether accounts are “prominently recognized,”^4^ suggesting that human biases may be easily interjected given that internal signals required for verification are not explicitly stated.

Our analysis has several limitations. The sample may exclude some verified PHAs that did not meet keyword-based selection criteria (eAppendix in the Supplement). Potentially confounding variables (eg, age and institutional affiliations) were not assessed.

Despite increasing numbers in medicine, women and other marginalized populations receive less respect and have lower rates of recognition and promotion.^6^ Differences in verified status among women and international users may suggest that these voices are also less valued in online discourse. This disparity may further propagate sex and other inequities among physicians.

## Notes

Supplement.
eAppendix.
