# Error in Author Name in Byline

**DOI:** 10.1001/jamanetworkopen.2022.40840

**Published:** 2022-10-18

In the Research Letter titled “Sex and Location Differences in Verification Status of Physician-Held Social Media Platform Accounts,”^1^ published August 8, 2022, there was an error in an author’s name in the byline. Brian Y. Chen’s name should have included the middle initial. This article has been corrected.^1^
