# Supplementary material for: Sex and Location Differences in Verification Status of Physician-Held Social Media Platform Accounts
Source: JAMA Netw Open. 2022 Aug 8;5(8):e2225671. doi: 10.1001/jamanetworkopen.2022.25671 (PMC9361082; doi:10.1001/jamanetworkopen.2022.25671)
Supplement: Supplement. — eAppendix. [file jamanetwopen-e2225671-s001.pdf]

Supplemental Online Content

Rupert D, Shah K, Chen B, et al. Sex and location differences in verification status of physician-held social media platform accounts. *JAMA Netw Open*. 2022;5(8):e2225671. doi:10.1001/jamanetworkopen.2022.25671

eAppendix.

This supplemental material has been provided by the authors to give readers additional information about their work.

© 2022 Rupert D et al. *JAMA Network Open.*

eAppendix

Supplementary Methods
Twitter accounts were accessed in November 2020 and manually assessed in July 2021. Keywords were used to identify physician users (see **Keywords** below).

Physician status and sex were manually confirmed by two independent researchers (FC, MS) using name, pronouns, and public profile images. Independent researchers were themselves of different sexes and academic rankings in order to balance potential biases. Accounts which were no longer verified at the time of analysis or inactive. Accounts for which sex could not be determined were excluded from sex-based analysis; 22 accounts were excluded. Accounts with unlisted location were excluded from location-based analysis; 67 accounts were excluded. Profile descriptors were classified as relating to politics, business, media, or “other” (arts, and athletics).

DDR conducted all statistical analyses using GraphPad Prism software (version 9.3.0).

This study met IRB exemption status per Memorial Sloan Kettering’s Institution Review Board given use of data publicly available on the internet. STROBE guidelines were used for study type verification and reporting. $P \leq .05$ was considered statistically significant.

Keywords to Identify Physicians

allergist
anesthesiologist
anesthesiology
Cancer doc
cardiologist
cardiology
cards
chief clinical officer
chief medical officer
crit care
critical care
D.O.
dermatologist
dermatology
doctor
em doc
em md
emergency doc
emergency med physician
emergency medicine

© 2022 Rupert D et al. *JAMA Network Open.*

emergency physician
ENT
er doc
er doctor
family doc
Family med
family medicine
family physician
gastroenterologist
gastroenterology
general internal medicine
general internist
genmed
geriatric medicine
geriatrician
GI doc
GYN
gynecologist
gynecology
Hem Onc
Hem/onc
hematologist
hematology
HemOnc
hospitalist
HPM
icu doc
id doc
infectious disease doctor
infectious disease physician
infectious diseases doctor
infectious diseases physician
infectiousdisease doc
infectiousdisease md
infectiousdiseases doc
infectiousdiseases md
intensivist
internal medicine
internist

© 2022 Rupert D et al. *JAMA Network Open.*

M.D.
MD
MD,
medical director
medical toxicology
MedOnc
nephrologist
nephrology
neurological surgery
neurologist
neurology
neurosurgeon
Neurosurgery
NSU
ob gyn
ob/gyn
obgyn
ob-gyn
obstetrician
obstetrics
obstetrics & gynecology
oncologist
oncology
ophthalmologist
ophthalmology
ophtho
ortho
orthopaedic
orthopaedic surgeon
orthopaedic surgery
orthopedic
orthopedic surgeon
orthopedic surgery
osteopathic medicine
osteopathic physician
osteopathy
otolaryngologist
otolaryngology
pain medicine

© 2022 Rupert D et al. *JAMA Network Open.*

Pain specialist
Palliative Care
Palliative Care
Palliative Doc
pathologist
pathology
pediatrician
pediatrics
peds
pgy
physical medicine & rehabilitation
physician
plastic surgery
plastics
plastics
pm & r
PM&R
PMNR
precision medicine
psych
psych
psychiatrist
psychiatry
pulm
Pulm/crit care
pulmonary
pulmonologist
pulmonology
radiation oncologist
radiation oncology
radiologist
radiology
radonc
radonc
rads
rads
resident
respiratory medicine
sleep medicine

© 2022 Rupert D et al. *JAMA Network Open.*

sports medicine
surgeon
surgery
surgonc
thoracic surgeon
thoracic surgery
TSU
Uro/gyn
urologist
urology
WIM
WomenInMedicine

© 2022 Rupert D et al. *JAMA Network Open.*
